# Supplementary material for: Spatial joint profiling of DNA methylome and transcriptome in tissues
Source: Nature. 2025 Sep 3;646(8087):1261–71. doi: 10.1038/s41586-025-09478-x (PMC12571926; doi:10.1038/s41586-025-09478-x)
Supplement: Supplementary file 1 — Supplementary Figs. 1–4 and Supplementary Tables 1–6. [file 41586_2025_9478_MOESM1_ESM.pdf]

nature portfolio

https://doi.org/10.1038/s41586-025-09478-x

Supplementary information

Spatial joint profiling of DNA methylome and transcriptome in tissues

In the format provided by the authors and unedited

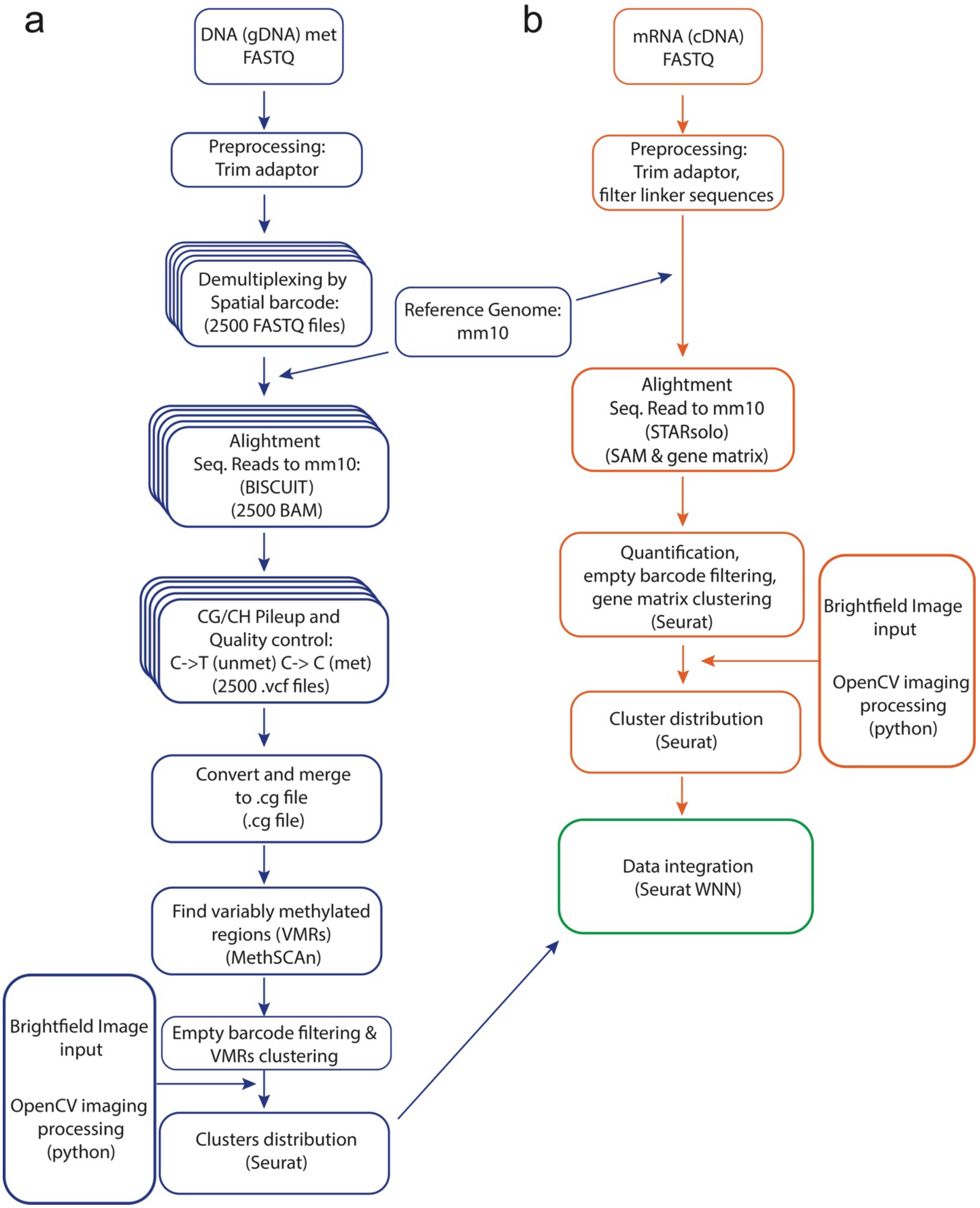

a
DNA (gDNA) met FASTQ
Preprocessing: Trim adaptor
Demultiplexing by Spatial barcode: (2500 FASTQ files)
Reference Genome: mm10
Alightment Seq. Reads to mm10: (BISCUIT) (2500 BAM)
CG/CH Pileup and Quality control: C->T (unmet) C-> C (met) (2500 .vcf files)
Convert and merge to .cg file (.cg file)
Find variably methylated regions (VMRs) (MethSCAn)
Brightfield Image input
OpenCV imaging processing (python)
Empty barcode filtering & VMRs clustering
Clusters distribution (Seurat)
b
mRNA (cDNA) FASTQ
Preprocessing: Trim adaptor, filter linker sequences
Alightment Seq. Read to mm10 (STARsolo) (SAM & gene matrix)
Quantification, empty barcode filtering, gene matrix clustering (Seurat)
Brightfield Image input
OpenCV imaging processing (python)
Cluster distribution (Seurat)
Data integration (Seurat WNN)

Supplementary Fig. 1: Computational workflow for Spatial-DMT data processing and analysis. The workflow diagram illustrates the steps involved in processing and analyzing spatial DNA methylation and RNA transcription data from Spatial-DMT. For DNA methylation, the process includes FASTQ preprocessing (trimming of adaptors and filtering of linker sequences), demultiplexing of spatial barcodes for 2500 FASTQ files, alignment of sequencing reads to the reference genome (e.g., mm10) using BISulfite-seq CUI Toolkit (BISCUIT) (resulting in 2500 BAM files), CG/CH pileup and quality control (variant calling to generate 2500 .vcf files and then conversion and merging to .cg files), identification of variably methylated regions (VMRs) using MethSCAn, empty barcode filtering and VMR clustering, and visualization of spatial clusters using Seurat, by integrating with brightfield images for spatial context processed by OpenCV. For mRNA data, the process includes FASTQ preprocessing (trimming of adaptors and filtering of linker sequences), alignment of sequencing reads to the reference genome (e.g., mm10) using STARsolo (generating SAM and gene matrix files), quantification of gene expression, filtering of empty barcodes, and visualization of spatial clusters using Seurat, by integrating with brightfield images for spatial context processed by OpenCV. The final step involves the integration of DNA methylation and RNA transcription data using Seurat WNN analysis to achieve integrated spatial multi-omics profiles.

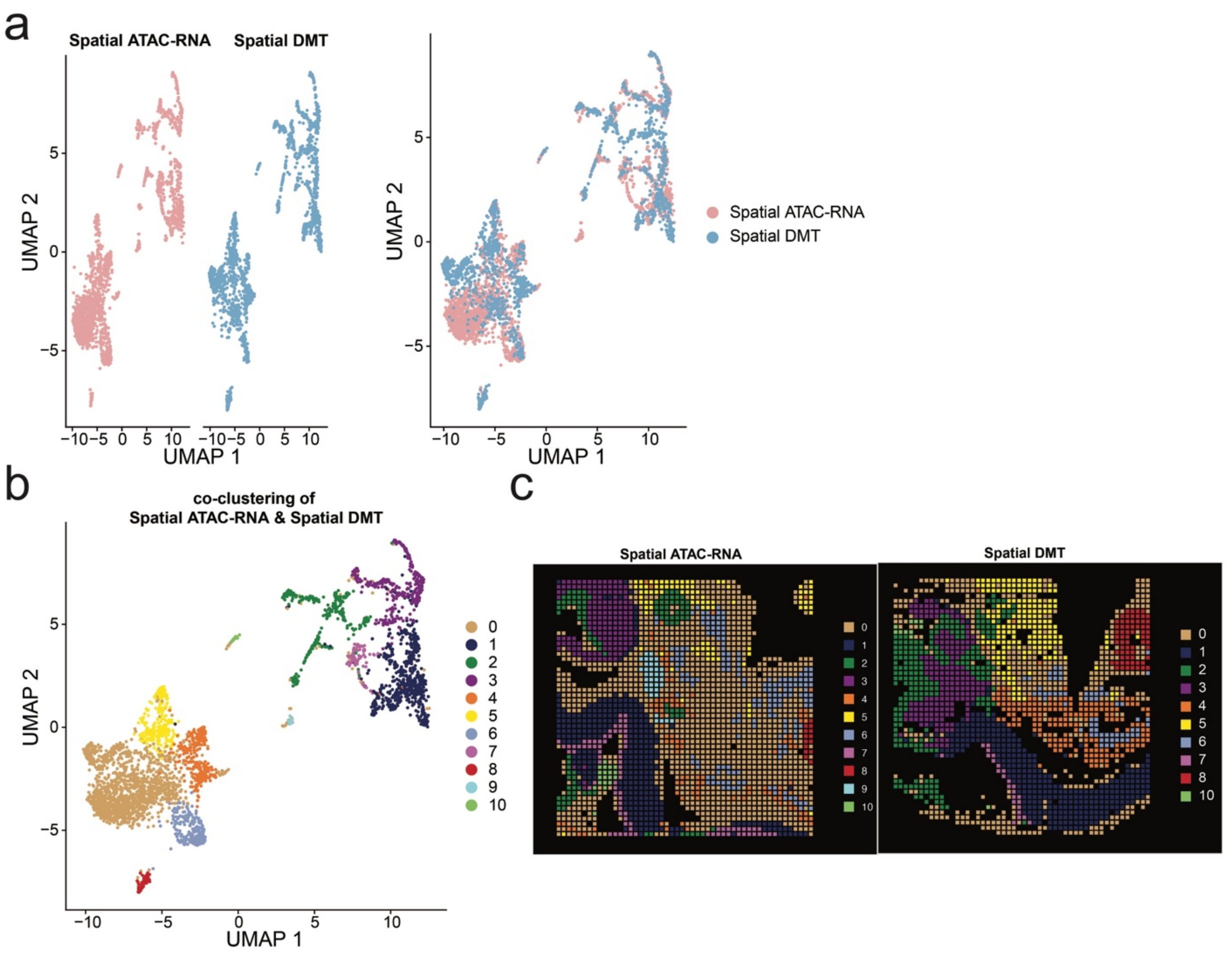

Supplementary Fig. 2: Integrative analysis of E13 embryo data. a-b, Integrated visualization of Spatial-DMT and spatial-ATAC-RNA-seq[2] datasets from E13 mouse embryos. Cells are colored according to experiments **(a)** and unsupervised co-clustering results **(b)**. **c,** Spatial distribution of co-clustering results, comparing spatial-ATAC-RNA-seq (left) and Spatial-DMT (right). Spatial maps show distinct clustering patterns, with each color representing the shared clusters defined from integrated analysis in **(b)**. Concordant spatial domains were shown across both technologies.

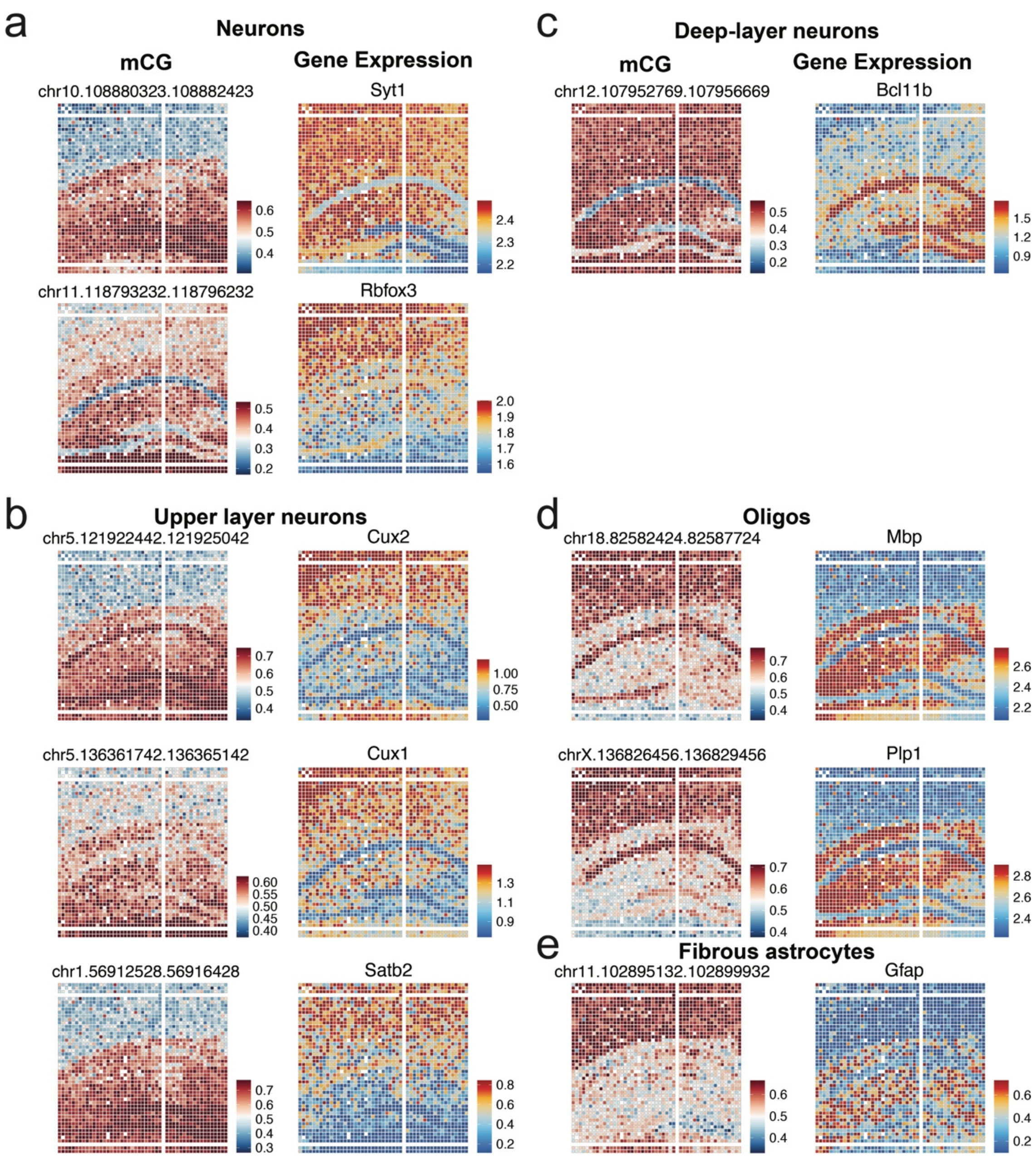

Supplementary Fig. 3: **Spatial mapping of DNA methylation and RNA expression levels of selected markers across distinct cell types in P21 mouse brain.** Heatmaps illustrating the spatial distribution of DNA methylation levels and RNA expression levels for markers of neurons (*Syt1*, *Rbfox3*) **(a)**, upper layer neurons (*Cux2*, *Cux1*, *Satb2*) **(b)**, deep-layer neurons (*Bcl11b*) **(c)**, oligos (*Mbp*, *Plp1*) **(d)**, and fibrous astrocytes (*Gfap*) **(e)**.

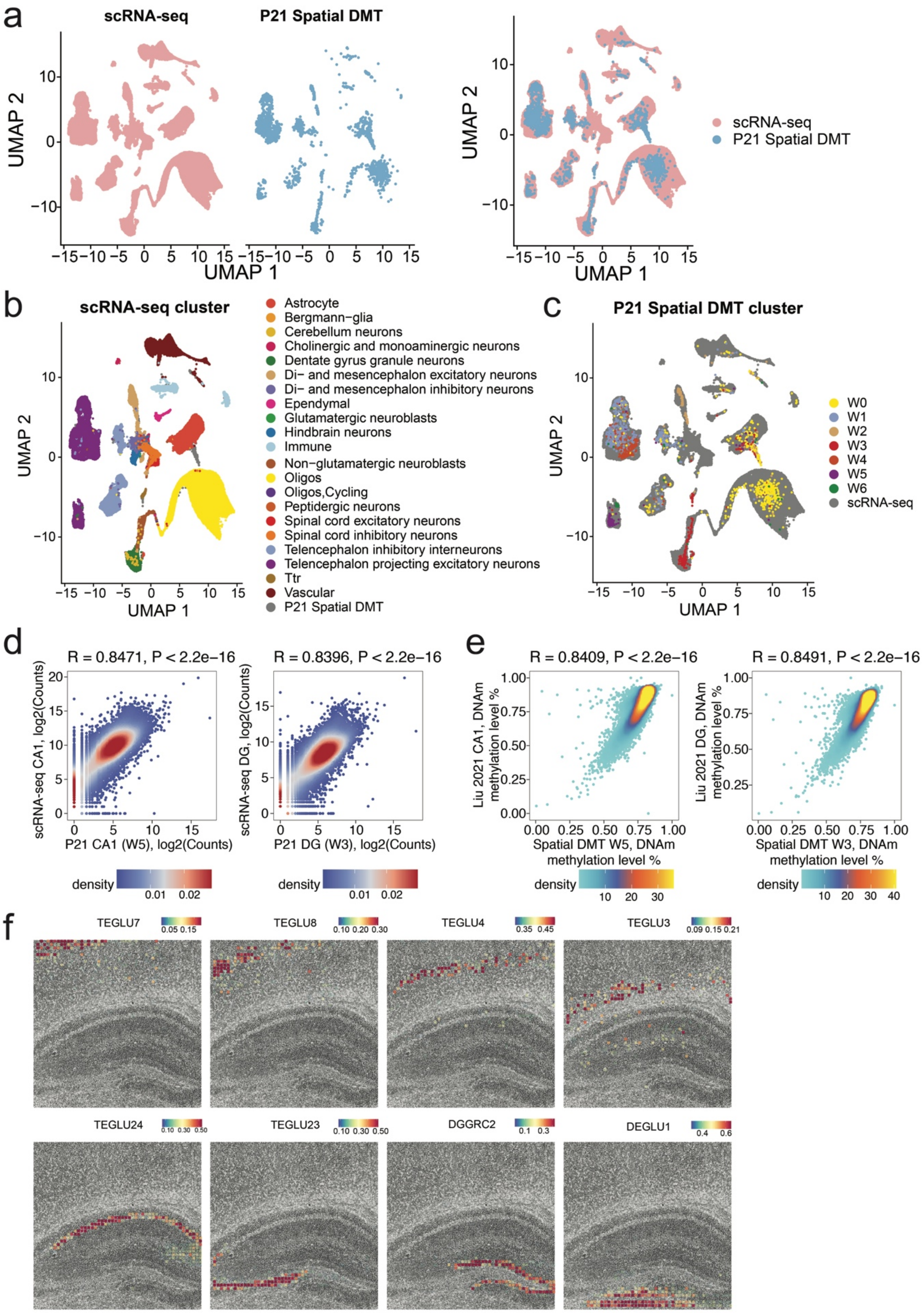

a
scRNA-seq
P21 Spatial DMT
UMAP 2
UMAP 1
scRNA-seq
P21 Spatial DMT
b
scRNA-seq cluster
Astrocyte
Bergmann−glia
Cerebellum neurons
Cholinergic and monoaminergic neurons
Dentate gyrus granule neurons
Di− and mesencephalon excitatory neurons
Di− and mesencephalon inhibitory neurons
Ependymal
Glutamatergic neuroblasts
Hindbrain neurons
Immune
Non−glutamatergic neuroblasts
Oligos
Oligos,Cycling
Peptidergic neurons
Spinal cord excitatory neurons
Spinal cord inhibitory neurons
Telencephalon inhibitory interneurons
Telencephalon projecting excitatory neurons
Ttr
Vascular
P21 Spatial DMT
c
P21 Spatial DMT cluster
W0
W1
W2
W3
W4
W5
W6
scRNA-seq
d
R = 0.8471, P < 2.2e−16
R = 0.8396, P < 2.2e−16
scRNA-seq CA1, log2(Counts)
P21 CA1 (W5), log2(Counts)
scRNA-seq DG, log2(Counts)
P21 DG (W3), log2(Counts)
density
e
R = 0.8409, P < 2.2e−16
R = 0.8491, P < 2.2e−16
Liu 2021 CA1, DNAm methylation level %
Spatial DMT W5, DNAm methylation level %
Liu 2021 DG, DNAm methylation level %
Spatial DMT W3, DNAm methylation level %
density
f
TEGLU7
TEGLU8
TEGLU4
TEGLU3
TEGLU24
TEGLU23
DGGRC2
DEGLU1

Supplementary Fig. 4: Integrative analysis of P21 mouse brain data. a-c, Integrated visualization of Spatial-DMT and scRNA-seq datasets from adolescent mouse brain[44]. Cells are colored according to experiments **(a)**, cell annotations from scRNA-seq data[44] **(b)**, and unsupervised clustering results from Spatial-DMT **(c)**. **d,** Correlation of gene expression profiles from cluster W5 (CA1) and W3 (DG) with a reference scRNA-seq dataset[44] with two-sided Pearson correlation test. **e,** Correlation of DNA methylation profiles (100kb bin) from cluster W5 (CA1) and W3 (DG) with a single-cell methylation dataset[5] with two-sided Pearson correlation test. **f,** Spatial distribution of selected cell types resolved by cell-type decomposition of spatial transcriptomic pixels using a reference scRNA-seq dataset[44], including cerebral cortex excitatory neurons (TEGLU7, TEGLU8, TEGLU4, TEGLU3), cornu ammonis excitatory neurons (TEGLU24 and TEGLU23), dentate gyrus granule neurons (DGGRC2), and thalamus excitatory neurons (DEGLU1).

Supplementary Table 1. DNA oligos for PCR and preparation of the sequencing library.

RT-primer	/5Phos/CATCGGCGTACGACTNNNNNNNNNN/iBiodT/TTTTTTTTTTTTTTVN
TSO	AAGCAGTGGTATCAACGCAGAGTGAATrGrG+G
SLS5ME-A-H10	/5AmMC12/TCGTCGGCAGCGTCAGATGTGTATAAGAGACAG(H:33330034)(H)(H)(H)(H)(H)(H)(H)(H)(H)(H)/3AmMO/
SLP5RC	/5Phos/CTGTCTCTTATACACATCTGACGCTGCCGACGA/3AmMO/
Primer 1	AAGCAGTGGTATCAACGCAGAGT
Primer 2	CAAGCGTTGGCTTCTCGCATCT
Ligation linker 1	AGTCGTACGCCGATGCGAAACATCGGCCAC
Ligation linker 2	CGAATGCTCTGGCCTCTCAAGCACGTGGAT
N501	AATGATACGGCGACCACCGAGATCTACACTAGATCGCTCGTCGGCAGCGTCAGATGTGTATAAGAGACAG
N702	CAAGCAGAAGACGGCATACGAGATCTAGTACGGTCTCGTGGGCTCGGAGATGTGTATAAGAGACAGCAAGCGTTGGCTTCTCGCATCT
N704	CAAGCAGAAGACGGCATACGAGATGCTCAGGAGTCTCGTGGGCTCGGAGATGTGTATAAGAGACAGCAAGCGTTGGCTTCTCGCATCT
N705	CAAGCAGAAGACGGCATACGAGATAGGAGTCCGTCTCGTGGGCTCGGAGATGTGTATAAGAGACAGCAAGCGTTGGCTTCTCGCATCT
N706-HT	CAAGCAGAAGACGGCATACGAGATCATGCCTAGTCTCGTGGGCTCGGAGATGTGTATAAGAGACAGTAAGTGTTGGTTTTTTGTATTT
N707-HT	CAAGCAGAAGACGGCATACGAGATGTAGAGAGGTCTCGTGGGCTCGGAGATGTGTATAAGAGACAGTAAGTGTTGGTTTTTTGTATTT
N708-HT	CAAGCAGAAGACGGCATACGAGATCCTCTCTGGTCTCGTGGGCTCGGAGATGTGTATAAGAGACAGTAAGTGTTGGTTTTTTGTATTT

Supplementary Table 2. Barcode A Sequence

Name	Sequence
Barcode A-1	/5Phos/AGGCCAGAGCATTCGAACGTGATGTGGCCGATGTTTCG
Barcode A-2	/5Phos/AGGCCAGAGCATTCGAAACATCGGTGGCCGATGTTTCG
Barcode A-3	/5Phos/AGGCCAGAGCATTCGATGCCTAAGTGGCCGATGTTTCG
Barcode A-4	/5Phos/AGGCCAGAGCATTCGAGTGGTCAGTGGCCGATGTTTCG
Barcode A-5	/5Phos/AGGCCAGAGCATTCGACCACTGTGTGGCCGATGTTTCG
Barcode A-6	/5Phos/AGGCCAGAGCATTCGACATTGGCGTGGCCGATGTTTCG
Barcode A-7	/5Phos/AGGCCAGAGCATTCGCAGATCTGGTGGCCGATGTTTCG
Barcode A-8	/5Phos/AGGCCAGAGCATTCGCATCAAGTGTGGCCGATGTTTCG
Barcode A-9	/5Phos/AGGCCAGAGCATTCGCGCTGATCGTGGCCGATGTTTCG
Barcode A-10	/5Phos/AGGCCAGAGCATTCGACAAGCTAGTGGCCGATGTTTCG
Barcode A-11	/5Phos/AGGCCAGAGCATTCGCTGTAGCCGTGGCCGATGTTTCG
Barcode A-12	/5Phos/AGGCCAGAGCATTCGAGTACAAGGTGGCCGATGTTTCG
Barcode A-13	/5Phos/AGGCCAGAGCATTCGAACAACCAGTGGCCGATGTTTCG
Barcode A-14	/5Phos/AGGCCAGAGCATTCGAACCGAGAGTGGCCGATGTTTCG
Barcode A-15	/5Phos/AGGCCAGAGCATTCGAACGCTTAGTGGCCGATGTTTCG
Barcode A-16	/5Phos/AGGCCAGAGCATTCGAAGACGGAGTGGCCGATGTTTCG
Barcode A-17	/5Phos/AGGCCAGAGCATTCGAAGGTACAGTGGCCGATGTTTCG
Barcode A-18	/5Phos/AGGCCAGAGCATTCGACACAGAAGTGGCCGATGTTTCG
Barcode A-19	/5Phos/AGGCCAGAGCATTCGACAGCAGAGTGGCCGATGTTTCG
Barcode A-20	/5Phos/AGGCCAGAGCATTCGACCTCCAAGTGGCCGATGTTTCG
Barcode A-21	/5Phos/AGGCCAGAGCATTCGACGCTCGAGTGGCCGATGTTTCG
Barcode A-22	/5Phos/AGGCCAGAGCATTCGACGTATCAGTGGCCGATGTTTCG
Barcode A-23	/5Phos/AGGCCAGAGCATTCGACTATGCAGTGGCCGATGTTTCG
Barcode A-24	/5Phos/AGGCCAGAGCATTCGAGAGTCAAGTGGCCGATGTTTCG
Barcode A-25	/5Phos/AGGCCAGAGCATTCGAGATCGCAGTGGCCGATGTTTCG
Barcode A-26	/5Phos/AGGCCAGAGCATTCGAGCAGGAAGTGGCCGATGTTTCG
Barcode A-27	/5Phos/AGGCCAGAGCATTCGAGTCACTAGTGGCCGATGTTTCG
Barcode A-28	/5Phos/AGGCCAGAGCATTCGATCCTGTAGTGGCCGATGTTTCG
Barcode A-29	/5Phos/AGGCCAGAGCATTCGATTGAGGAGTGGCCGATGTTTCG
Barcode A-30	/5Phos/AGGCCAGAGCATTCGCAACCACAGTGGCCGATGTTTCG

Barcode A-31	/5Phos/AGGCCAGAGCATTCGGACTAGTAGTGGCCGATGTTTCG
Barcode A-32	/5Phos/AGGCCAGAGCATTCGCAATGGAAGTGGCCGATGTTTCG
Barcode A-33	/5Phos/AGGCCAGAGCATTCGCACTTCGAGTGGCCGATGTTTCG
Barcode A-34	/5Phos/AGGCCAGAGCATTCGCAGCGTTAGTGGCCGATGTTTCG
Barcode A-35	/5Phos/AGGCCAGAGCATTCGCATACCAAGTGGCCGATGTTTCG
Barcode A-36	/5Phos/AGGCCAGAGCATTCGCCAGTTCAGTGGCCGATGTTTCG
Barcode A-37	/5Phos/AGGCCAGAGCATTCGCCGAAGTAGTGGCCGATGTTTCG
Barcode A-38	/5Phos/AGGCCAGAGCATTCGCCGTGAGAGTGGCCGATGTTTCG
Barcode A-39	/5Phos/AGGCCAGAGCATTCGCCTCCTGAGTGGCCGATGTTTCG
Barcode A-40	/5Phos/AGGCCAGAGCATTCGCGAACTTAGTGGCCGATGTTTCG
Barcode A-41	/5Phos/AGGCCAGAGCATTCGCGACTGGAGTGGCCGATGTTTCG
Barcode A-42	/5Phos/AGGCCAGAGCATTCGCGCATACAGTGGCCGATGTTTCG
Barcode A-43	/5Phos/AGGCCAGAGCATTCGCTCAATGAGTGGCCGATGTTTCG
Barcode A-44	/5Phos/AGGCCAGAGCATTCGCTGAGCCAGTGGCCGATGTTTCG
Barcode A-45	/5Phos/AGGCCAGAGCATTCGCTGGCATAGTGGCCGATGTTTCG
Barcode A-46	/5Phos/AGGCCAGAGCATTCGGAATCTGAGTGGCCGATGTTTCG
Barcode A-47	/5Phos/AGGCCAGAGCATTCGCAAGACTAGTGGCCGATGTTTCG
Barcode A-48	/5Phos/AGGCCAGAGCATTCGGAGCTGAAGTGGCCGATGTTTCG
Barcode A-49	/5Phos/AGGCCAGAGCATTCGGATAGACAGTGGCCGATGTTTCG
Barcode A-50	/5Phos/AGGCCAGAGCATTCGGCCACATAGTGGCCGATGTTTCG

Supplementary Table 3. Barcode B Sequence

Name	Sequence
Barcode B-1	CAAGCGTTGGCTTCTCGCATCTAACGTGATATCCACGTGCTTGAG
Barcode B-2	CAAGCGTTGGCTTCTCGCATCTAAACATCGATCCACGTGCTTGAG
Barcode B-3	CAAGCGTTGGCTTCTCGCATCTATGCCTAAATCCACGTGCTTGAG
Barcode B-4	CAAGCGTTGGCTTCTCGCATCTAGTGGTCAATCCACGTGCTTGAG
Barcode B-5	CAAGCGTTGGCTTCTCGCATCTACCACTGTATCCACGTGCTTGAG
Barcode B-6	CAAGCGTTGGCTTCTCGCATCTACATTGGCATCCACGTGCTTGAG
Barcode B-7	CAAGCGTTGGCTTCTCGCATCTCAGATCTGATCCACGTGCTTGAG
Barcode B-8	CAAGCGTTGGCTTCTCGCATCTCATCAAGTATCCACGTGCTTGAG
Barcode B-9	CAAGCGTTGGCTTCTCGCATCTCGCTGATCATCCACGTGCTTGAG
Barcode B-10	CAAGCGTTGGCTTCTCGCATCTACAAGCTAATCCACGTGCTTGAG
Barcode B-11	CAAGCGTTGGCTTCTCGCATCTCTGTAGCCATCCACGTGCTTGAG
Barcode B-12	CAAGCGTTGGCTTCTCGCATCTAGTACAAGATCCACGTGCTTGAG
Barcode B-13	CAAGCGTTGGCTTCTCGCATCTAACAACCAATCCACGTGCTTGAG
Barcode B-14	CAAGCGTTGGCTTCTCGCATCTAACCGAGAATCCACGTGCTTGAG
Barcode B-15	CAAGCGTTGGCTTCTCGCATCTAACGCTTAATCCACGTGCTTGAG
Barcode B-16	CAAGCGTTGGCTTCTCGCATCTAAGACGGAATCCACGTGCTTGAG
Barcode B-17	CAAGCGTTGGCTTCTCGCATCTAAGGTACAATCCACGTGCTTGAG
Barcode B-18	CAAGCGTTGGCTTCTCGCATCTACACAGAAATCCACGTGCTTGAG
Barcode B-19	CAAGCGTTGGCTTCTCGCATCTACAGCAGAATCCACGTGCTTGAG
Barcode B-20	CAAGCGTTGGCTTCTCGCATCTACCTCCAAATCCACGTGCTTGAG
Barcode B-21	CAAGCGTTGGCTTCTCGCATCTACGCTCGAATCCACGTGCTTGAG
Barcode B-22	CAAGCGTTGGCTTCTCGCATCTACGTATCAATCCACGTGCTTGAG
Barcode B-23	CAAGCGTTGGCTTCTCGCATCTACTATGCAATCCACGTGCTTGAG
Barcode B-24	CAAGCGTTGGCTTCTCGCATCTAGAGTCAAATCCACGTGCTTGAG
Barcode B-25	CAAGCGTTGGCTTCTCGCATCTAGATCGCAATCCACGTGCTTGAG
Barcode B-26	CAAGCGTTGGCTTCTCGCATCTAGCAGGAAATCCACGTGCTTGAG
Barcode B-27	CAAGCGTTGGCTTCTCGCATCTAGTCACTAATCCACGTGCTTGAG
Barcode B-28	CAAGCGTTGGCTTCTCGCATCTATCCTGTAATCCACGTGCTTGAG
Barcode B-29	CAAGCGTTGGCTTCTCGCATCTATTGAGGAATCCACGTGCTTGAG
Barcode B-30	CAAGCGTTGGCTTCTCGCATCTCAACCACAATCCACGTGCTTGAG

Barcode B-31	CAAGCGTTGGCTTCTCGCATCTGACTAGTAATCCACGTGCTTGAG
Barcode B-32	CAAGCGTTGGCTTCTCGCATCTCAATGGAAATCCACGTGCTTGAG
Barcode B-33	CAAGCGTTGGCTTCTCGCATCTCACTTCGAATCCACGTGCTTGAG
Barcode B-34	CAAGCGTTGGCTTCTCGCATCTCAGCGTTAATCCACGTGCTTGAG
Barcode B-35	CAAGCGTTGGCTTCTCGCATCTCATACCAAATCCACGTGCTTGAG
Barcode B-36	CAAGCGTTGGCTTCTCGCATCTCCAGTTCAATCCACGTGCTTGAG
Barcode B-37	CAAGCGTTGGCTTCTCGCATCTCCGAAGTAATCCACGTGCTTGAG
Barcode B-38	CAAGCGTTGGCTTCTCGCATCTCCGTGAGAATCCACGTGCTTGAG
Barcode B-39	CAAGCGTTGGCTTCTCGCATCTCCTCCTGAATCCACGTGCTTGAG
Barcode B-40	CAAGCGTTGGCTTCTCGCATCTCGAACTTAATCCACGTGCTTGAG
Barcode B-41	CAAGCGTTGGCTTCTCGCATCTCGACTGGAATCCACGTGCTTGAG
Barcode B-42	CAAGCGTTGGCTTCTCGCATCTCGCATACAATCCACGTGCTTGAG
Barcode B-43	CAAGCGTTGGCTTCTCGCATCTCTCAATGAATCCACGTGCTTGAG
Barcode B-44	CAAGCGTTGGCTTCTCGCATCTCTGAGCCAATCCACGTGCTTGAG
Barcode B-45	CAAGCGTTGGCTTCTCGCATCTCTGGCATAATCCACGTGCTTGAG
Barcode B-46	CAAGCGTTGGCTTCTCGCATCTGAATCTGAATCCACGTGCTTGAG
Barcode B-47	CAAGCGTTGGCTTCTCGCATCTCAAGACTAATCCACGTGCTTGAG
Barcode B-48	CAAGCGTTGGCTTCTCGCATCTGAGCTGAAATCCACGTGCTTGAG
Barcode B-49	CAAGCGTTGGCTTCTCGCATCTGATAGACAATCCACGTGCTTGAG
Barcode B-50	CAAGCGTTGGCTTCTCGCATCTGCCACATAATCCACGTGCTTGAG

Supplementary Table 4. Chemicals and reagents

Name	Catalog number	Vender
Formaldehyde solution	PI28906	Thermo Fisher Scientific
Glycine	50046	Sigma-Aldrich
0.1N HCl	2104-50ML	Sigma-Aldrich
NaCl	AM9760G	Thermo Fisher Scientific
$MgCl_2$	AM9530G	Thermo Fisher Scientific
Digitonin	G9441	Promega
Sodium dodecyl sulfate	71736	Sigma-Aldrich
HEPES pH 7.5	BBH-75-250	Boston BioProducts
EDTA Solution pH 8.0	AB00502	AmericanBio
Bovine Serum Albumin (BSA)	A8806	Sigma-Aldrich
NP40	11332473001	Sigma-Aldrich
Triton X-100	T8787	Sigma-Aldrich
T4 DNA Ligase	M0202L	New England Biolabs
T4 DNA Ligase Reaction Buffer	B0202S	New England Biolabs
NEBuffer 3.1	B7203S	New England Biolabs
DPBS	14190144	Thermo Fisher Scientific
Proteinase K	EO0491	Thermo Fisher Scientific
NEBNext High-Fidelity 2X PCR Master Mix	M0541L	New England Biolabs
SYBR Green I Nucleic Acid Gel Stain	S7563	Thermo Fisher Scientific
DNA Clean & Concentrator-5	D4014	Zymo Research
Tn5 Transposase - unloaded	C01070010	Diagenode
Tagmentation Buffer (2x)	C01019043	Diagenode
Maxima H Minus Reverse Transcriptase (200 U/μl)	EP0751	Thermo Fisher Scientific
VeraSeq Ultra DNA polymerase	7520L	Qiagen
dNTP mix	R0192	Thermo Fisher Scientific
SUPERase•In™ RNase Inhibitor (RI)	AM2694	Thermo Fisher Scientific
RNase Inhibitor (40 U/μl)	Y9240L	Enzymatics
SPRI beads	A63880	Beckman Coulter
Dynabeads MyOne C1	65001	Thermo Fisher Scientific
Kapa Hotstart HiFi ReadyMix	KK2601	Kapa Biosystems
NEBNext® Enzymatic Methyl-seq Conversion Module	E7125S	New England Biolabs
Nextera XT DNA Library Preparation Kit	FC-131-1024	Illumina

Supplementary Table 5. Summary of metrics for DNA methylation and RNA transcription co-profiling of all samples.

Sample	E11 (10 μm)		E11#1 (50 μm)		E11#2 (50 μm)	
Modality	DNA	RNA	DNA	RNA	DNA	RNA
Total raw reads	2,756,840,516	172,492,101	2,864,519,160	98,539,436	2,863,772,201	350,172,191
Total pixels	2493	2493	1954	1954	1947	1947
Retained read %	32.20	70.71	65.72	64.78	61.89	68.06
Retained reads (before alignment)	887,671,712	121,967,091	1,882,630,968	63,833,555	1,772,395,274	238,318,000
Ave. retained reads / pixel	355,069	12,476	753,052	7,544	708,958	37,810
Total # of genes	-	25,118	-	25,992	-	27,306
Ave. gene # / pixel	-	1,890	-	2,417	-	4,626
Ave. UMI # / pixel	-	3,596	-	5,108	-	16,709
Ave. mCG % coverage	0.62	-	1.09	-	1.29	-
Ave. mCG # / pixel	136,639	-	238,040	-	281,447	-
Ave. mCH % coverage	-	-	-	-	-	-
Ave. mCH # / pixel	-	-	-	-	-	-

Sample	E13 (50 μm)		P21 (20 μm)	
Modality	DNA	RNA	DNA	RNA
Total raw reads	3,018,600,847	180,508,321	3,914,623,812	110,320,779
Total pixels	1699	1699	2235	2235
Retained read %	55.69	72.65	42.48	73.88
Retained reads (before alignment)	1,680,936,562	131,147,184	1,662,805,699	81,501,569
Ave. retained reads / pixel	672,375	15,715	665,122	11,205
Total # of genes	-	28,695	-	23,822
Ave. gene # / pixel	-	3,788	-	2,525
Ave. UMI # / pixel	-	9,830	-	5,796
Ave. mCG % coverage	1.17	-	0.89	-
Ave. mCG # / pixel	255,402	-	194,224	-
Ave. mCH % coverage	-	-	0.43	-
Ave. mCH # / pixel	-	-	4,773,740	-

Supplementary Table 6. Summary of significant chromHMM full stack annotations.

Name	Description
mEnhA2	Active enhancers in most cell and tissue types (e.g. brain, heart, digestive, limb, epithelium) weaker in some cell types (e.g. liver, spleen, immune). Strong DNase, ATAC, H3K4me1, H3K27ac, H3K9ac.
mEnhA4	Active enhancers signals (DNase, H3K4me1, H3K27ac, ATAC) in multiple tissue types including kidney, lung, heart, stomach, limb.
mEnhA5	Weak to moderate enhancer signal (H3K4me1, H3K27ac, DNase, ATAC) in multiple tissues including heart, stomach, kidney, intestine, lung, and liver.
mEnhA7	Strong H3K4me1 and DNase in brain, epithelium/neural tube, limb, and embryo. Lower H3K4me1 and DNase in others.
mEnhA8	H3K4me1 and DNase in brain and epithelium/neural tube, also low H3K4me1 and DNase in various other tissues.
mEnhA9	Enhancers in brain and epithelium/neural tube; H3K4me1, H3K27ac, DNase, ATAC in those tissue.
mEnhA10	Strong enhancers in brain and epithelium/neural tube with H3K4me1, H3K27ac, H3K9ac, DNase, ATAC in those tissues.
mEnhA12	Active enhancer signals (ATAC, DNase, H3K4me1, H3K27ac) in many tissue types. Weaker or less active enhancer signals in some tissue types including brain, epithelium/neural tube, connective tissue, and embryo.
mEnhA16	Active enhancer mark (H3K27ac) strongest in limb, embryo. Moderate DNase in a subset of other cell types (limb, epithelium, musculature, ESC, gonad, adrenal gland, stomach, intestine, kidney, heart, brain).
mPromF4	Promoter state enriched flanking TSS. Stronger H3K4me2 than H3K4me1 and H3K4me3. Low to moderate ATAC, DNase, H3K4me1/2/3, H3K9ac,
mPromF7	Poised promoters/proximal enhancers particularly in brain and epithelium/neural tube; Strongest signals for H3K4me2 in Brain and Neural tubes and higher relative expression in those tissues;
openC1	DNase is the strongest mark in state and found varying levels for different cell and tissue types. Some weak H3K27me3 and H3K4me1.
openC2	Weak DNase in various cell types. Weaker H3K4me1 and H3K27me3 in a subset.
