# Supplementary material for: Spatial joint profiling of DNA methylome and transcriptome in tissues
Source: Nature. 2025 Sep 3;646(8087):1261–71. doi: 10.1038/s41586-025-09478-x (PMC12571926; doi:10.1038/s41586-025-09478-x)
Supplement: Supplementary file 2 — Reporting Summary [file 41586_2025_9478_MOESM2_ESM.pdf]

nature portfolio

Corresponding author(s): Yanxiang Deng

Last updated by author(s): 07/15/2025

Reporting Summary

Nature Portfolio wishes to improve the reproducibility of the work that we publish. This form provides structure for consistency and transparency in reporting. For further information on Nature Portfolio policies, see our Editorial Policies and the Editorial Policy Checklist.

Statistics

For all statistical analyses, confirm that the following items are present in the figure legend, table legend, main text, or Methods section.

n/a	Confirmed	
☐	☒	The exact sample size (n) for each experimental group/condition, given as a discrete number and unit of measurement
☐	☒	A statement on whether measurements were taken from distinct samples or whether the same sample was measured repeatedly
☐	☒	The statistical test(s) used AND whether they are one- or two-sided<br>*Only common tests should be described solely by name; describe more complex techniques in the Methods section.*
☐	☒	A description of all covariates tested
☐	☒	A description of any assumptions or corrections, such as tests of normality and adjustment for multiple comparisons
☐	☒	A full description of the statistical parameters including central tendency (e.g. means) or other basic estimates (e.g. regression coefficient) AND variation (e.g. standard deviation) or associated estimates of uncertainty (e.g. confidence intervals)
☐	☒	For null hypothesis testing, the test statistic (e.g. F, t, r) with confidence intervals, effect sizes, degrees of freedom and P value noted<br>*Give P values as exact values whenever suitable.*
☒	☐	For Bayesian analysis, information on the choice of priors and Markov chain Monte Carlo settings
☒	☐	For hierarchical and complex designs, identification of the appropriate level for tests and full reporting of outcomes
☐	☒	Estimates of effect sizes (e.g. Cohen's d, Pearson's r), indicating how they were calculated

Our web collection on statistics for biologists contains articles on many of the points above.

Software and code

Policy information about availability of computer code

Data collection	Keyence Imaging System BZ-X800, Illumina NovaSeq 6000 and NovaSeq X Plus system.
Data analysis	STARsolo (version 2.7.10b), BISCUIT (version 0.3.14), Seurat package (version 5.1.0), MethSCAn (version 1.0.0), FigR package (version 0.1.0), HOMER (version 4.11), GenomicRanges (version 4.4), clusterProfiler (version 4.2), R (version 4.3.1), RStudio (version 2024.04.0), knowYourCG (version 1.3.15), ggalluvial (version 0.12.5), pheatmap (1.0.12), BZ-X800 1.1.2.4, Slingshot v2.2.1.<br><br>The data analysis pipeline and code to reproduce analyses are available on GitHub (https://github.com/zhou-lab/Spatial-DMT-2024/).

For manuscripts utilizing custom algorithms or software that are central to the research but not yet described in published literature, software must be made available to editors and reviewers. We strongly encourage code deposition in a community repository (e.g. GitHub). See the Nature Portfolio guidelines for submitting code & software for further information.

Data

Policy information about availability of data

All manuscripts must include a data availability statement. This statement should provide the following information, where applicable:

- Accession codes, unique identifiers, or web links for publicly available datasets
- A description of any restrictions on data availability
- For clinical datasets or third party data, please ensure that the statement adheres to our policy

Raw and processed data reported in this paper are deposited in the Gene Expression Omnibus (GEO) with accession code GSE270498. Published data for data quality comparison and integrative data analysis include single cell atlas of mouse embryos (https://oncoscape.v3.sttrcancer.org/atlas.gs.washington.edu.mouse.rna/downloads, https://omg.gs.washington.edu/), mouse brain atlas (http://mousebrain.org/adolescent/downloads.html), and Allen Mouse Brain Atlas (https://developingmouse.brain-map.org/).

Research involving human participants, their data, or biological material

Policy information about studies with human participants or human data. See also policy information about sex, gender (identity/presentation), and sexual orientation and race, ethnicity and racism.

Reporting on sex and gender	N/A
Reporting on race, ethnicity, or other socially relevant groupings	N/A
Population characteristics	N/A
Recruitment	N/A
Ethics oversight	N/A

Note that full information on the approval of the study protocol must also be provided in the manuscript.

Field-specific reporting

Please select the one below that is the best fit for your research. If you are not sure, read the appropriate sections before making your selection.

☒ Life sciences ☐ Behavioural & social sciences ☐ Ecological, evolutionary & environmental sciences

For a reference copy of the document with all sections, see nature.com/documents/nr-reporting-summary-flat.pdf

Life sciences study design

All studies must disclose on these points even when the disclosure is negative.

Sample size	No directly relevant. No sample size calculation was performed. Samples sizes were chosen primarily based on experiment length, sequencing costs. The current manuscript mainly described a new method for profiling spatially resolved DNA methylation and Transcription, the sample sizes are sufficient because each sample serves as a proof-of-concept for the new technology.
Data exclusions	No data were excluded from the study.
Replication	All attempts at replication was successful. For E11 mouse embryo, replicate experiments have been done on adjacent tissue sections to test the reproducibility of the new technology. Other experiments were performed once to serve as a proof-of-concept for the new technology.
Randomization	Randomization was not applicable because the focus of this paper is the development of a new spatial multiomics technology for profiling spatially resolved DNA methylation and transcription, it did not involve allocating samples/organisms/participants into experimental groups.
Blinding	Blinding was not applicable because the focus of this paper is the development of a new spatial multiomics technology for profiling spatially resolved DNA methylation and transcription, it did not involve group allocation, and by extension, blinding.

Reporting for specific materials, systems and methods

We require information from authors about some types of materials, experimental systems and methods used in many studies. Here, indicate whether each material, system or method listed is relevant to your study. If you are not sure if a list item applies to your research, read the appropriate section before selecting a response.

Materials & experimental systems

n/a	Involved in the study	
☒	☐	Antibodies
☒	☐	Eukaryotic cell lines
☒	☐	Palaeontology and archaeology
☐	☒	Animals and other organisms
☒	☐	Clinical data
☒	☐	Dual use research of concern
☒	☐	Plants

Methods

n/a	Involved in the study	
☒	☐	ChIP-seq
☒	☐	Flow cytometry
☒	☐	MRI-based neuroimaging

Animals and other research organisms

Policy information about studies involving animals; ARRIVE guidelines recommended for reporting animal research, and Sex and Gender in Research

Laboratory animals	All mice used were on C57BL/6 background. Animal were maintained in 12 h light/12 h dark cycle at room temperatures ranging between 20-25°C and humidities between 40-60%. P21 mouse was used in Spatial-DMT for the co-profiling of DNA methylation and Transcription.
Wild animals	No wild animals were used in this study.
Reporting on sex	Sex was not important for this study since the tissues were used to benchmark a new genomics protocol, which we anticipate would provide identical results regardless of sex.
Field-collected samples	No field collected sample were used in this study.
Ethics oversight	Juvenile mouse brain tissue (P21) was obtained from the C57BL/6 mice housed in the University of Pennsylvania Animal Care Facilities under pathogens-free conditions. All procedures used were pre-approved by the Institutional Animal Care and Use Committee.

Note that full information on the approval of the study protocol must also be provided in the manuscript.

Plants

Seed stocks	No seed was use in this study
Novel plant genotypes	N/A
Authentication	N/A
